# Paraganglioma of the greater omentum: Case report and review of the literature

**DOI:** 10.1186/1477-7819-5-87

**Published:** 2007-08-03

**Authors:** Fotios Archontovasilis, Haridimos Markogiannakis, Christina Dikoglou, Panagiotis Drimousis, Konstantinos G Toutouzas, Dimitrios Theodorou, Stilianos Katsaragakis

**Affiliations:** 1 1^st ^Department of Propaedeutic Surgery, Hippokrateion Hospital, Athens Medical School, University of Athens, Q. Sofias 114 avenue, 11527 Athens, Greece; 2 Histopathology Department, Hippokrateion Hospital, Q. Sofias 114 avenue, 11527 Athens, Greece

## Abstract

**Background:**

Extra-adrenal, intra-abdominal paraganglioma constitutes a rare neoplasm and, moreover, its location in the greater omentum is extremely infrequent.

**Case presentation:**

A 46-year-old woman with an unremarkable medical history presented with an asymptomatic greater omentum mass that was discovered incidentally during ultrasonographic evaluation due to menstrual disturbances. Clinical examination revealed a mobile, non-tender, well-circumscribed mass in the right upper and lower abdominal quadrant. Blood tests were normal. Contrast-enhanced abdominal computed tomography (CT) scan confirmed a huge (15 × 15 cm), well-demarcated, solid and cystic, heterogeneously enhanced mass between the right liver lobe and right kidney. Exploratory laparotomy revealed a large mass in the greater omentum. The tumor was completely excised along with the greater omentum. Histopathology offered the diagnosis of benign greater omentum paraganglioma. After an uneventful postoperative course, the patient was discharged on the 4^th ^postoperative day. She remains free of disease for 2 years as appears on repeated CT scans as well as magnetic resonance imaging (MRI) and scintigraphy performed with radiotracer-labeled metaiodobenzyl-guanidine (MIBG) scans.

**Conclusion:**

This is the second reported case of greater omentum paraganglioma. Clinical and imaging data of patients with extra-adrenal, intra-abdominal paragangliomas are variable while many of them may be asymptomatic even when the lesion is quite large. Thorough histopathologic evaluation is imperative for diagnosis and radical excision is the treatment of choice. Since there are no definite microscopic criteria for the distinction between benign and malignant tumors, prolonged follow-up is necessary.

## Background

Extra-adrenal paragangliomas of the sympathoadrenal neuroendocrine system consist 5%–15% of sporadic pheochromocytomas and are located anywhere extending from the upper neck to the pelvic floor, parallel to the autonomic nervous system [1,2]. Extra-adrenal, abdominal paragangliomas are divided into three groups: superior para-aortic, inferior para-aortic, and urinary bladder tumors [2]. Rarely, extra-adrenal paragangliomas can occur aberrantly outside this distribution. The annual worldwide incidence of paragangliomas, as well as in Greece, is approximately 1/300,000 [3,4].

Herein, we report such a case of a paraganglioma occurring in the greater omentum in a 46-year-old woman along with a review of the literature. To the best of our knowledge, this is the second reported case of greater omentum paraganglioma in the literature [5]. Apart from its extremely infrequent location, the reported case is important because of the rather huge diameter of the tumor and the asymptomatic course of the patient prior to diagnosis as well as the characteristic radiological and pathological figures that are presented.

## Case presentation

**Figure 1:**
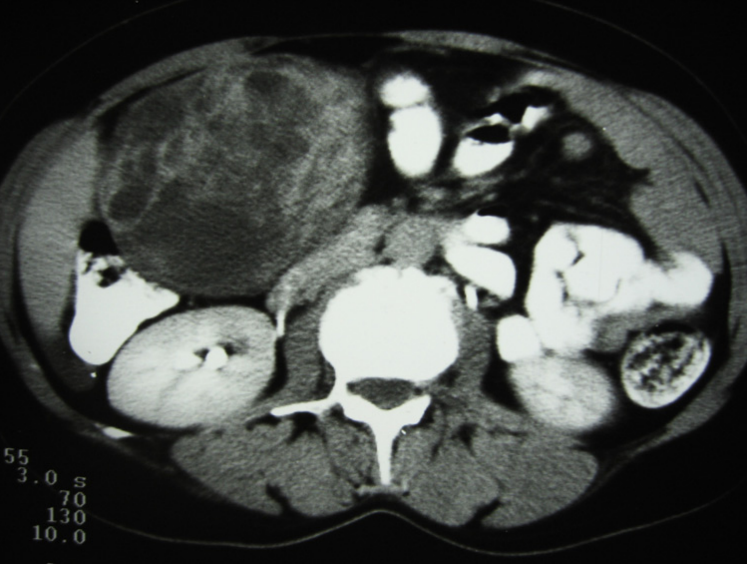
Contrast-enhanced abdominal CT scan, with oral and i.v. contrast, shows a large (15 × 15 cm), well-demarcated, solid and cystic, heterogeneously enhanced mass located between the right lobe of the liver and the right kidney.

**Figure 2:**
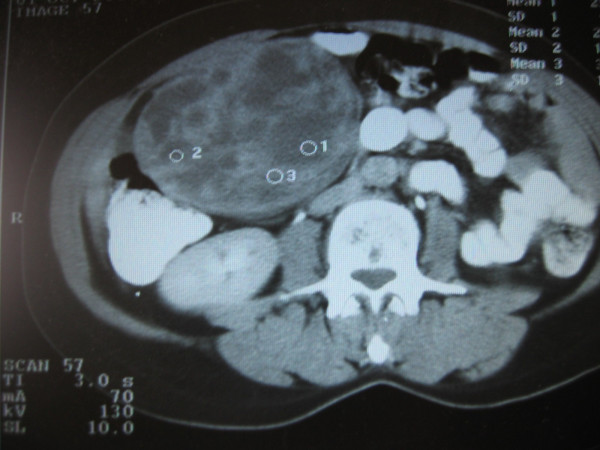
Contrast enhanced CT scan showing heterogeneously enhanced mass located above right kidney.

A 46-year-old woman with an unremarkable medical history presented with an asymptomatic greater omentum mass incidentally found during ultrasonographic evaluation due to menstrual disturbances. Clinical examination showed a non-tender, mobile, well-circumscribed mass in the right upper and lower abdominal quadrant. Esophagogastroduodenoscopy revealed mild extrinsic compression of the stomach and duodenum. Contrast-enhanced abdominal computed tomography (CT) scan, with oral and i.v. contrast, confirmed a large (15 × 15 cm), well-demarcated, solid and cystic, heterogeneously enhanced mass between the right liver lobe and right kidney (Figures 1 and 2). Blood tests were normal, including levels of serous neoplasmatic markers such as carbohydrate antigen 19-9, carcinoembryonic antigen, carbohydrate antigen 125, and a-feto-protein, as well as anti-echinoccocal antibodies. Additionally, although no clinical or imaging data suggested a pheochromocytoma, due to the close anatomic relation of the tumor to the right kidney and adrenal gland, blood levels of catecholamines, plasma-free metanephrines, aldosterone, and rennin along with 24-hour urine levels of total catecholamines and their metabolic products (vanillylmandelic acid and metanephrines) were measured and found to be within normal limits. Due to the huge size of the mass, diagnostic laparoscopy was considered impossible and, therefore, not performed. Exploratory laparotomy revealed a large, encapsulated, cystic and solid mass in the greater omentum without any lymph node or distant metastasis. The tumor was completely excised along with the greater omentum.

**Figure 3:**
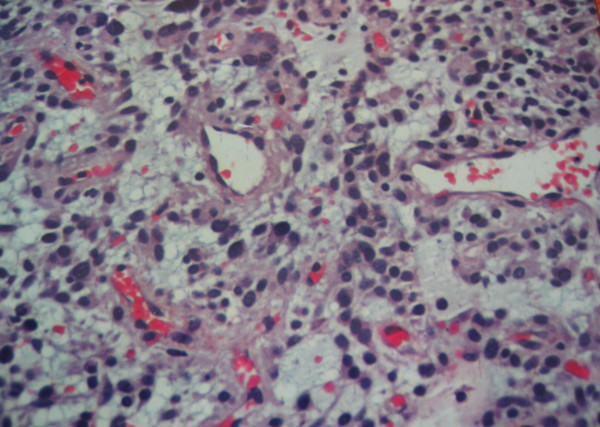
Photomicrograph showing paraganglioma with nuclear pleomorphism and hyperchromasia (HE × 20).

**Figure 4:**
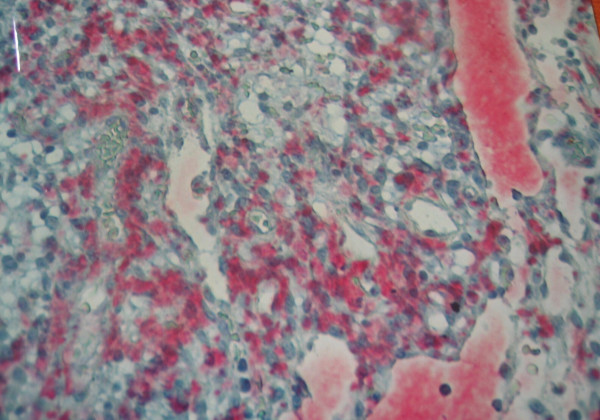
Photomicrograph of paraganglioma showing positive immunostaining for chromogranin A. Streptavidin-alkaline phosphatase reaction (× 20).

On histopathologic evaluation, the tumor contained small round cells (chief cells) arranged in well defined nests, having a mixture of trabecular and alveolar pattern. These groups of cells were separated by fibrovascular septa, giving a characteristic "zellballen" nested pattern. The tumor cells were polyhedral, with round or oval nuclei, and contained intracytoplasmic hyaline globules; no cytologic atypia or mitosis was observed, although there were some nuclear pleomorphisms (Figure 3). No local invasion, vascular invasion, confluent tumor necrosis or coarse nodularity was identified. Immunohistochemically, the tumor cells revealed positive immunostaining for chromogranin A and S-100 protein (Figure 4). Additionally, positive staining for [Leu^5^]-enkephalin, [Met^5^]-enkephalin, somatostatin, pancreatic polypeptide, and vasoactive intestinal polypeptide (VIP) was identified. A few nuclei were immunoreactive in Ki-67/MIB-1 but proliferative activity as the percentage of Ki-67 immunoreactive cells (proliferative index) was <1% while immunostaining for cytokeratin, c-kit, and CD34 was negative. No telomerase activity (TA), human telomerase reverse transcriptase expression (hTERT: telomerase catalytic subunit), topoisomerase IIa expression, or mRNA expression of matrix metalloproteinase (MMP)-2 and EMMPRIN (extracellular matrix metalloproteinase inducer) was detected. Based on histologic and immunohistochemical features, a diagnosis of benign greater omentum paraganglioma was offered.

Due to histopathologic diagnosis, genetic testing for familial paraganglioma, neurofibromatosis type 1, von Hippel-Lindau disease, the Carney triad, multiple endocrine neoplasia type 2, and mutations of the succinate dehydrogenase genes (SDHB, SDHC, and SDHD) was performed that was negative.

After an uneventful postoperative course, the patient was discharged on the 4^th ^postoperative day. She remains free of disease for 2 years as appears from repeated biochemical testing (plasma catecholamines, plasma-free metanephrines, urinary catecholamines, urinary vanillylmandelic acid, urinary total metanephrines, urinary fractionated metanephrines), CT scans as well as magnetic resonance imaging (MRI) and scintigraphy performed with radiotracer-labeled metaiodobenzyl-guanidine (MIBG) scans.

## Discussion

The paraganglion system is formed by numerous collections of neuroepithelial cells. Their common morphologic characteristic is the presence of numerous cytoplasmic neurosecretory granules containing catecholamines. Paraganglioma is the generic term applied to tumors of paraganglia regardless of location. Paragangliomas of the adrenal medulla, the most common site of paragangliomas, are known as pheochromocytomas while those located outside the adrenal gland as extra-adrenal pheochromocytomas [6].

Extra-adrenal paraganglia are divided into two categories: those related to the parasympathetic system and those connected with the orthosympathetic system. The former, usually nonchromaffin, lie in the head and neck including the carotid body, intravagal, jugulotympanic, and mediastinal and aorticopulmonary paraganglia [7,8]. The latter are chromaffin, associated with the peripheral sympathetic nervous system, and secrete catecholamines in response to sympathetic neural stimulation. They lie in the paraaxial region of the trunk close to the paravertebral and prevertebral ganglia or in the connective tissue adjacent to pelvic organs, predominating along the thoracolumbar paraaortic region in the retroperitoneum [9,10]. The adrenal medulla and the organs of Zuckerkandl are the only ones visible to the naked eye [6,10].

Extra-adrenal, intra-abdominal paragangliomas are divided into three groups: superior para-aortic, inferior para-aortic, and urinary bladder paragangliomas [9,11]. Rarely, paragangliomas have been described in other unusual sites, such as the gallbladder [12], mesentery [13], kidney [14], prostate [15], and ovary [16]. We hypothesize that the paraganglioma in our patient was derived from the paraganglionic cells by vertebral migration from the root of superior mesenteric artery. The development of a paraganglioma in the greater omentum is extremely rare and this case represents the second report in the literature [5]. As with other paragangliomas described outside the distribution of paraganglionic tissue, our case may be explained by the migration patterns of the widely dispersed neural crest cells.

Paragangliomas may be hereditary and can be associated with familial paraganglioma, neurofibromatosis type 1, von Hippel-Lindau disease, the Carney triad, multiple endocrine neoplasia type 2, and mutations of the succinate dehydrogenase genes (SDHB, SDHC, and SDHD) [1]. Genetic testing should, therefore, be considered in all patients with paraganglioma. In the presented case, genetic testing was performed and was negative.

Histologically, paraganglia contain nests of neuroendocrine cells surrounded by sustenacular cells. This pattern is most clearly seen in parasympathetic paraganglia as the characteristic "zellballen". In sympathetic paraganglia, neuroendocrine cells are referred to as chromaffin cells and in parasympathetic as chief cells. These cells are immunopositive for neuroendocrine markers including synaptophysin and chromogranin A and for several neuropeptides. Features that may predict malignancy include extra-adrenal location, confluent tumor necrosis, vascular invasion, local invasion, coarse nodularity, and absence of hyaline globules [17]. Moreover, decreased expression of neuropeptides and, particularly, negative staining for enkephalins, somatostatin, pancreatic polypeptide, and VIP have been associated with malignancy [18]. Although the presented tumor was extra-adrenal, the presence of hyaline globules and the absence of confluent tumor necrosis, vascular invasion, local invasion, and coarse nodularity along with the positive staining for the above-mentioned polypeptides show the benign nature of this lesion.

Most patients with extra-adrenal, abdominal paragangliomas present with abdominal pain or a palpable abdominal mass [19]; however, they may be asymptomatic even when the lesion is large and the tumor is incidentally found during radiographic evaluation for other reasons, such as the presented patient. Other symptoms may be nausea, vomiting, diarrhea, abdominal distension, and weight loss. Increased catecholamine secretion in functional tumors is responsible for symptoms such as hypertension, flashing, sweating, headache, diaphoresis, anxiety, tachycardia, or palpitations [9,11,19].

Even though imaging studies are helpful, diagnosis of extra-adrenal, intra-abdominal paragangliomas can be safely done only with careful histologic and immunohistochemical evaluation. Contrast-enhanced abdominal CT scan is useful for diagnosis; however, no CT feature unique for paragangliomas has been found [11]. Abdominal MRI and MIBG are essential in localization and characterization of paragangliomas while MRI has the highest sensitivity in detection of extra-adrenal paragangliomas as well as pheochromocytomas [20,21].

The majority of abdominal tumors are benign; 10%–50% of intra-abdominal, extra-adrenal paragangliomas are reported to be malignant [9-11,19,22]. Malignant forms frequently develop distant metastases in lung, liver, and lymph nodes. Annual postoperative biochemical testing (plasma catecholamines, plasma-free metanephrines, urinary catecholamines, urinary vanillylmandelic acid, urinary total metanephrines, and urinary fractionated metanephrines), CT scans as well as MRI and MIBG scans are essential for assessment for metastatic disease, tumor recurrence or delayed appearance of multiple primary tumors [1].

Clinical and histological distinction between benign and malignant cases is difficult [9,23,24]. The only proof of malignancy is the presence of metastases or local invasion while in localized tumors there are no absolute criteria for predicting malignant potential. This principle has the drawback that some cases will initially be classified as benign and then reclassified as malignant if metastases are identified at follow-up. There is, therefore, a clear need for additional diagnostic tools to enable detection of malignant cases at initial surgery. Additional diagnostic markers would be valuable, but, up to date, no reliable prognostic markers have been identified [23,24]. Although several markers such as immunohistochemical expression of the cell proliferation marker Ki-67/MIB-1, telomerase activity, hTERT expression, topoisomerase IIa expression, and mRNA expression of MMP-2 and EMMPRIN metalloproteinases have shown promising results, they need further evaluation. Even though these markers seem to be very valuable, particularly when combined with histopathology, no single test or combination of tests has yielded sufficiently high sensitivity and specificity to result in widespread acceptance in everyday clinical practice [22-26]. Due to the rarity of the tumor, multicenter studies are probably necessary in this effort [24]. The very low proliferation index (<1%) found in the presented case and the fact that no telomerase activity, hTERT expression, topoisomerase IIa expression, or mRNA expression of MMP-2 and EMMPRIN metalloproteinases was identified show the benign nature of this lesion.

The treatment of choice for paragangliomas is complete surgical resection. Traditional treatment consists of open exploration and resection. However, laparoscopic management is nowadays increasingly being used for their management due to the advances in laparoscopic technique and refined preoperative imaging even though few studies regarding the laparoscopic treatment of extra-adrenal intra-abdominal paragangliomas exist in the literature because of the rarity of this clinical entity [1]. Intraoperative ultrasound is considered an invaluable tool for laparoscopic surgeons in these cases. In these studies of laparoscopic management, encouraging results are presented; it should be mentioned, however, that all tumors were less than 4 cm [1]. In the presented patient, due to the huge size of the tumor, laparoscopy was considered impossible and, therefore, not performed.

Since clinical presentation and imaging data of these patients are variable and non specific, histological distinction between benign and malignant cases as well as prediction of malignant potential are difficult, and local recurrence or distant metastases are not infrequent, a multidisciplinary approach regarding effective diagnosis, management and follow-up is essential and should involve the close collaboration of endocrinologists, surgeons, anesthesiologists, geneticists, laboratory specialists, radiologists, oncologists, and pathologists.

## Conclusion

Paraganglioma of the greater omentum is extremely infrequent. Recognition of this tumor as a cause of an abdominal mass is important. Clinical and imaging data of patients with extra-adrenal, intra-abdominal paragangliomas are highly variable and non specific while many of them may be asymptomatic even when the lesion is quite large, such as the presented case. Thorough histological and immunohistochemical evaluation is, therefore, imperative and the only safe modality for diagnosis. The first line treatment is surgical resection, while chemotherapy and radiotherapy seem to be useless in benign lesions [19,22]. Since there are no definite microscopic criteria for the distinction between benign and malignant tumors, radical excision is the treatment of choice and prolonged follow-up is necessary.

## Competing interests

The author(s) declare that they have no competing interests.

## Authors' contributions

FA contributed to manuscript conception, research, acquisition of data, drafting and writing of the manuscript. HM contributed to manuscript conception, research, acquisition of data, drafting and writing of the manuscript. CD carried out the histopathologic evaluation and contributed to writing of the manuscript. PD assisted in the operation and contributed to organising and drafting of the manuscript. KGT assisted in the operation and critically revised the manuscript. DT assisted in the operation and critically revised the manuscript. SK carried out the operation and contributed to acquisition of consent and critical review of the manuscript.

All authors read and approved the final manuscript.
